# Lower serum PRL is associated with the development of non-alcoholic fatty liver disease: a retrospective cohort study

**DOI:** 10.1186/s12876-022-02619-w

**Published:** 2022-12-16

**Authors:** Ping Xu, Ye Zhu, Xinlu Ji, Huayang Ma, Pengzi Zhang, Yan Bi

**Affiliations:** 1 Department of Endocrinology, Nanjing Drum Tower Hospital Clinical College of Nanjing Medical University, Nanjing, China; 2 Department of Endocrinology, Drum Tower Hospital Affiliated to Nanjing University Medical School, Branch of National Clinical Research Centre for Metabolic Diseases, Nanjing, China; 3 Endocrine and Metabolic Disease Medical Center, Drum Tower Hospital Affiliated to Nanjing University Medical School, Nanjing, China

**Keywords:** NAFLD, Prolactin, Abdominal ultrasonography, Longitudinal study, Gender, Predictor

## Abstract

**Background:**

Non-alcoholic fatty liver disease (NAFLD) has become an epidemic worldwide and has been linked to a series of metabolic co-morbidities. Prolactin (PRL) has recently been found to have a negative effect on NAFLD, but a causal relationship is not well-understood. Here we investigated the causative relationship between PRL and NAFLD occurrence.

**Methods:**

In this retrospective cohort study, we enrolled patients without NAFLD who were diagnosed by abdominal ultrasonography undergone serum PRL testing at 8.00 a.m. at baseline, and followed up for a median of 32 (19, 46) months.

**Results:**

This study enrolled 355 persons [215 men and 140 women; media age 56 (49, 64) years], in which 72 (20.28%) patients who eventually developed NAFLD. Compared with those in the non-NAFLD group, basal serum PRL levels of patients were lower in the NAFLD group [male: 7.35 (5.48, 10.60) vs. 9.13 (6.92, 12.50) ug/L, *P* = 0.002; female: 5.66 (4.67, 9.03) vs. 9.01 (6.31, 11.60) ug/L, *P* = 0.009]. The prevalence of NAFLD was significantly decreased along with the increased quartile of basal serum PRL levels in both genders (*P* < 0.05). Serum PRL concentration was independently associated with NAFLD development [male: OR, 0.881 (0.777, 0.998), *P* = 0.047; female: OR, 0.725 (0.554, 0.949), *P* = 0.019].

**Conclusion:**

Our study is the first to find that basal serum PRL level can predict the occurrence of NAFLD and it may be a potential biomarker to prevent and treat NAFLD.

**Supplementary Information:**

The online version contains supplementary material available at 10.1186/s12876-022-02619-w.

## Introduction

Non-alcoholic fatty liver disease (NAFLD) has become one of the most prevalent causes of chronic liver disease worldwide [1]. According to estimated, the global prevalence of NAFLD was 25% [2], and the number of people with NAFLD will continue to grow by 30% by 2030 [3]. It comprises extensive histopathologic features ranging from simple steatosis to steatohepatitis, as well as progressing to liver cirrhosis and hepatocellular carcinoma eventually [4, 5]. And also, NAFLD significantly increases the risk of metabolic diseases and extra hepatic cancers, such as obesity, dyslipidemia, type 2 diabetes, gastro-intestinal cancers and bladder cancer [6–10]. Early screening and prevention of NAFLD helps to reduce the risk of NAFLD and its associated commodities.

Prolactin (PRL) is a hormone secreted predominantly by the anterior pituitary gland [11]. In addition to its well-known lactogenic action, PRL is now also known to act as a metabolic hormone [12]. It has been reported that lower serum PRL levels are associated with impaired glucose regulation and type 2 diabetes [13–15]. Importantly, we previously reported that lower serum PRL concentrations were associated with the presence of biopsy-diagnosed NAFLD in a cross-sectional study [16]. However, the cause-effect relationship between serum PRL concentrations and NAFLD occurrence is not well-understood.

Therefore, in the present study, we sought to explore the causative relationship between serum PRL concentrations and NAFLD occurrence in a retrospective cohort study. We aimed to corroborate whether basal circulating PRL level is an independent predictor of NAFLD occurrence. The findings of this study may have clinical implications for the management of NAFLD.

## Materials and methods

### Subject information

We performed this retrospective cohort study in the Endocrinology Department of Drum Tower Hospital affiliated to Nanjing University Medical School. Patients who went for regular health check-ups between July 2012 and July 2021 were enrolled in the study. Patients without NAFLD who were diagnosed by abdominal ultrasonography undergone serum PRL testing at 8.00 a.m. at baseline were included to investigate the correlation of serum PRL levels with the risk of incident NAFLD. Patients with any of the following conditions were excluded: history of alcohol consumption (≥ 140 g per week for male and ≥ 70 g per week for female) [17], chronic virus hepatitis, history of steatogenic agents (e.g. tetracycline), autoimmune hepatitis, type 1 diabetes mellitus (T1DM), pituitary disease, hyperprolactinemia, acromegaly, Cushing's syndrome, primary aldosteronism, pheochromocytoma, pregnancy and lactation, malignant tumors, hyperthyroidism or hypothyroidism, and acute inflammatory diseases. We considered the date of baseline ultrasound measurement to be the start date of follow-up, and the date of the last ultrasound measurement as the end date of follow-up. The protocol of the present study conformed to the guidelines of the Declaration of Helsinki and was approved by the Ethics Committee of Nanjing Drum Tower Hospital (2021-388-01).

### Clinical diagnosis for NAFLD

In all patients included, abdominal ultrasound was conducted under fasting conditions. Abdominal ultrasonography was used to diagnose NAFLD by Philips HD15 Ultrasound Unit (Netherlands). The scanning was done by the same group of qualified sonographers who were blinded to the patient’s clinical data using a standardized protocol. Clinical NAFLD is diagnosed through abdominal ultrasound by examining the characteristic echo patterns, including “bright liver”; increased echo contrast between hepatic and renal parenchyma; echo attenuation into the deep hepatic portion; vessel blurring or poor visualization of diaphragm [18].

### Clinical variables and biochemical measurements

We reviewed the electronic medical records of all patients in order to collect demographic and biochemical variables retrospectively. Body weight, height, waist circumference (WC), systolic blood pressure (SBP) and diastolic blood pressure (DBP) were measured by a qualified assessor. Body mass index (BMI) was assessed as body weight (kg) divided by height squared (m^2^).

Blood specimens of each individual were collected for laboratory analysis between 8:00 and 10:00 am after overnight fasting for at least 8 h. HbA1c and fasting blood glucose (FBG) were measured by high-performance liquid chromatography (HLC-73G8, Tosoh, Japan) and the hexokinase method (TBA-200FR, Tokyo, Japan), respectively. Fasting insulin (FINS) was determined via electrochemiluminescence immunoassay (Roche, USA). The homeostasis model assessment (HOMA) for IR was calculated using the following formula: fasting blood glucose (FBG, mmol/L) × fasting insulin (FINS, μIU/ml)/22.5. Triglycerides (TG), total cholesterol (TC), high-density lipoprotein cholesterol (HDL-C), low-density lipoprotein cholesterol (LDL-C), alanine aminotransferase (ALT), aspartate transaminase (AST), and creatinine (Cr), uric acid (UA), estimated glomerular filtration rate (eGFR) were measured through an autoanalyzer (Abbott Laboratories, Parsippany, USA). Thyroid stimulating hormone (TSH) was detected using electrochemiluminescence immunoassay (Cobase601, Roche, Swit). PRL was determined by an automated chemiluminescent microparticle immunoassay (Siemens Immulite 2000, UK).

### Statistical analysis

Because of discrepancies in serum PRL levels and NAFLD incidence between males and females, demographic and laboratory indicators for the two sexes were analyzed separately.

Normally distributed variables were reported as means ± standard deviation (SD) and analyzed by Student’s t-tests. Non-normally distributed variables were expressed as medians (25th, 75th percentiles) and analyzed by Mann–Whitney U-tests. Chi-square tests were applied to calculate the composition of rate.

Multivariate logistic regression analyses were used to determine the associations between PRL level and incident NAFLD. Serum PRL level was treated as a continuous variable.

Date management and statistical analyses were performed with SPSS Statistics software version 26.0 (IBM SPSS Inc., Chicago, USA). A two-sided *P*-value < 0.05 was considered statistically significant.

## Results

### Basal clinical parameters of patients in males and females

**Fig. 1:**
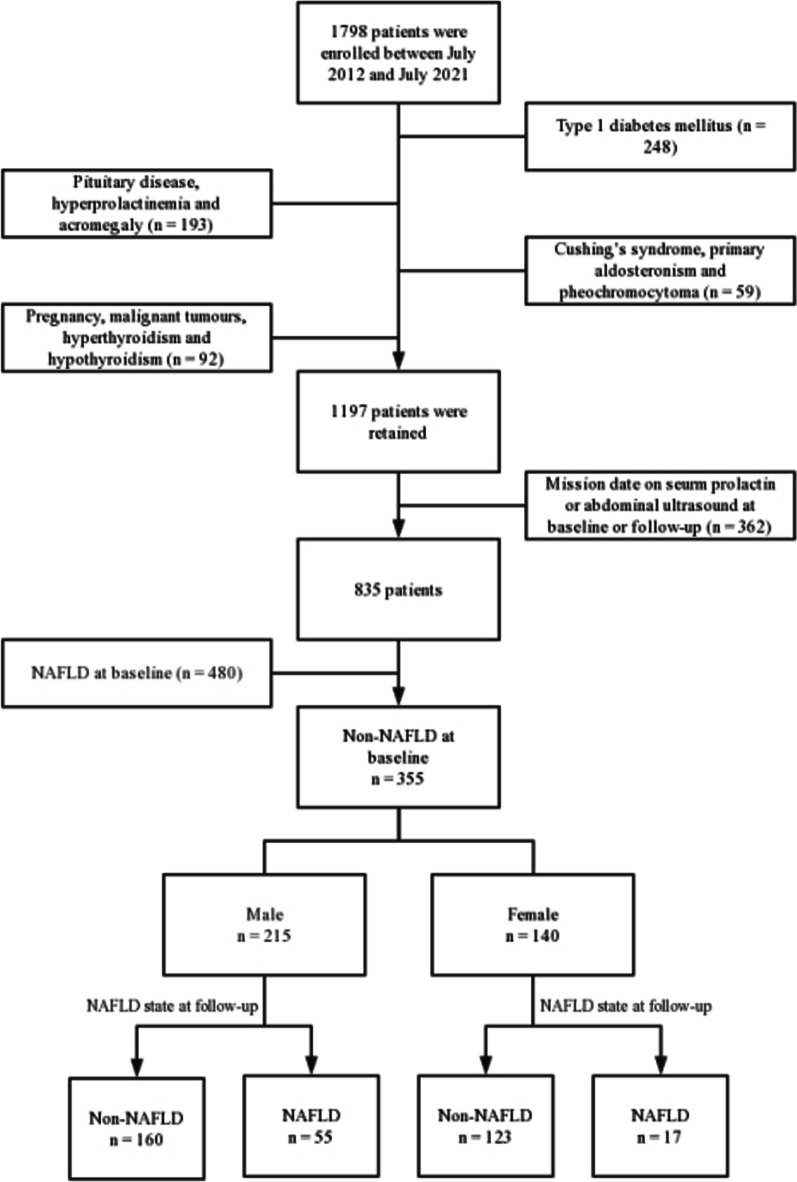
Flow chart of study population. A total of 1798 patients who were treated twice or more at Nanjing Drum Tower Hospital between July 2012 and July 2021 were initial enrolled. Finally, 355 patients were retained to explore the association of serum PRL levels with Risk of NAFLD

A total of 355 patients were enrolled, in which 215 were males and 140 were females (Fig. 1). The median age was 56 (49, 64) years, and the median BMI was 23.92 (21.91, 26.12) kg/m^2^. Over a median 32 (19, 46) months of follow-up, 72 patients developed NAFLD, the morbidity of NAFLD during follow-up was 20.28%. According to the status of their follow-up endpoint, the patients were divided into the non-NAFLD and NAFLD groups. The clinical characteristics of the 355 individuals at baseline and at follow-up are shown in Additional file 1: Table S1.

Male patients in the NAFLD group had significantly higher HOMA-IR, TG and TC (all *P*-value < 0.05) at baseline than those in non-NAFLD group, while no significant differences were found between the NAFLD and non-NAFLD groups among female patients (Table 1).

**Table 1:** Basal clinical parameters of patients with or without development of NAFLD at follow-up

Variables	Male			Female		
	NAFLD state at follow-up		*P*-value	NAFLD state at follow-up		*P*-value
	Non-NAFLD n = 160	NAFLD n = 55		Non-NAFLD n = 123	NAFLD n = 17	
Age (year)	58 (50, 66)	53 (45, 60)	0.003	56 (45, 65)	53 (49, 61)	0.385
BMI (kg/m^2^)	23.84 (22.05, 25.40)	24.22 (22.35, 26.23)	0.241	23.75 (20.45, 26.59)	26.56 (23.46, 29.40)	0.067
WC (cm)	90 (85, 95)	93 (86, 98)	0.174	83 (78, 96)	91 (79, 99)	0.294
Follow-up (month)	32 (21, 45)	34 (22, 50)	0.333	30 (14, 43)	34 (14, 50)	0.540
Diabetes, n (%)	158 (98.8%)	50 (90.9%)	0.005	94 (76.4%)	14 (82.4%)	0.585
Hypertension, n (%)	83 (51.9%)	31 (56.4%)	0.565	65 (52.8%)	7 (41.2%)	0.367
Hyperlipidemia, n (%)	40 (25.0%)	23 (41.8%)	0.018	31 (25.2%)	7 (41.2%)	0.165
SBP (mmHg)	133 (120, 144)	130 (118, 139)	0.129	133 (121, 148)	126 (104, 149)	0.191
DBP (mmHg)	78 (71, 86)	78 (71, 89)	0.462	78 (69, 86)	70 (63, 91)	0.368
HbA1c (%)	7.90 (6.75, 10.00)	7.60 (6.30, 10.90)	0.297	7.05 (6.00, 9.23)	8.50 (5.50, 10.90)	0.559
FBG (mmol/L)	7.08 (5.58, 8.94)	6.97 (5.47, 8.85)	0.932	5.98 (4.89, 8.39)	6.44 (4.68, 10.60)	0.753
HOMA-IR	1.47 (0.81, 3.00)	2.04 (1.42, 3.20)	0.032	1.84 (1.01, 3.97)	1.79 (1.21, 3.98)	0.925
TG (mmol/L)	1.07 (0.77, 1.50)	1.53 (1.03, 2.04)	0.001	1.03 (0.68, 1.42)	1.25 (0.85, 1.90)	0.084
TC (mmol/L)	4.04 (3.56, 4.80)	4.45 (3.59, 5.48)	0.019	4.43 (3.76, 5.22)	4.46 (4.15, 5.46)	0.515
HDL-C (mmol/L)	1.07 (0.84, 1.28)	0.97 (0.84, 1.21)	0.240	1.19 (0.98, 1.57)	1.15 (1.05, 1.37)	0.506
LDL-C (mmol/L)	2.30 (1.78, 2.80)	2.55 (1.77, 3.21)	0.086	2.47 (1.98, 3.04)	2.77 (2.26, 3.38)	0.258
ALT (U/L)	17.60 (13.85, 24.23)	17.40 (14.05, 28.00)	0.347	17.80 (13.70, 24.80)	23.90 (13.50, 33.25)	0.415
AST (U/L)	17.20 (14.90, 20.50)	18.60 (14.15, 22.05)	0.872	18.90 (15.70, 22.10)	17.80 (14.50, 23.85)	0.790
Cr (umol/L)	66.00 (60.00, 76.00)	65.00 (58.50, 74.00)	0.250	50.00 (43.00, 57.20)	48.00 (40.50, 57.50)	0.607
UA (umol/L)	318.50 (267.25, 372.00)	328.00 (287.50, 412.50)	0.050	270.00 (226.50, 312.75)	290.00 (247.00, 331.50)	0.544
eGFR (ml/min/1.73 m^2^)	116.46 (100.05, 130.16)	116.30 (99.24, 140.18)	0.596	116.69 (103.09, 142.47)	130.61 (100.99, 168.35)	0.427
TSH (mIU/L)	1.78 (1.34, 2.60)	1.83 (1.13, 2.63)	0.904	2.20 (1.44, 3.19)	1.98 (1.45, 3.22)	0.878
PRL (ug/L)	9.13 (6.92, 12.50)	7.35 (5.48, 10.60)	0.002	9.01 (6.31, 11.60)	5.66 (4.67, 9.03)	0.009

The data are expressed as mean ± standard deviation or median (interquartile range)

*NAFLD* Non-alcoholic fatty liver disease; *BMI* Body mass index; *WC* Waist circumference; *SBP* Systolic blood pressure; *DBP* Diastolic blood pressure; *FBG* Fasting blood glucose; *HOMA* Homeostasis model assessment; *TG* Triglycerides; *TC* Total cholesterol; *HDL-C* High-density lipoprotein cholesterol; *LDL-C* Low-density lipoprotein cholesterol; *ALT* Alanine aminotransferase; *AST* Aspartate transaminase; *Cr* Creatinine; *UA* Uric acid; *eGFR* Estimated glomerular filtration rate; *TSH* Thyroid stimulating hormone; *PRL* Prolactin

In males, basal serum PRL concentration was significantly lower in the NAFLD group than those in the non-NAFLD group [7.35 (5.48, 10.60) vs. 9.13 (6.92, 12.50) ug/L, *P* = 0.002]. Similarly, female patients in the NAFLD group also had lower serum PRL concentration [5.66 (4.67, 9.03) vs. 9.01 (6.31, 11.60) ug/L, *P* = 0.009] than the non-NAFLD patients.

### Decreased incidence of NAFLD grouped by quartiles of increased basal PRL

Based on the quartiles of serum PRL concentrations at baseline, the patients were divided into four groups. The quartile ranges of Q1, Q2, Q3, and Q4 of serum PRL levels were < 6.65, 6.65–8.68, 8.68–11.90, and > 11.90 ug/L in males and < 5.69, 5.69–8.75, 8.75–11.28, and > 11.28 ug/L in females, respectively. The clinical characteristics of each group of patients are shown in Additional file 1: Table S2.

**Fig. 2:**
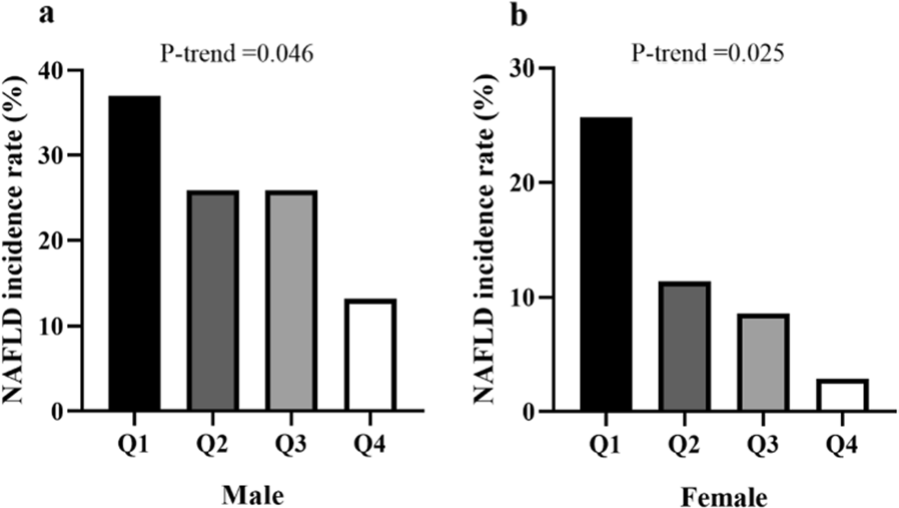
Incidence rate (%) of non-alcoholic fatty liver disease (NAFLD) by quartiles of the serum prolactin (PRL) levels at baseline. The quartile ranges of Q1, Q2, Q3, and Q4 of serum PRL levels were < 6.65, 6.65–8.68, 8.68–11.90, and > 11.90 ug/L in males and < 5.69, 5.69–8.75, 8.75–11.28, and > 11.28 ug/L in females. **Panel a** shows that the incidence rate of NAFLD decreased with the increase of quartile of PRL concentration in male patients (*P*—trend = 0.046). **Panel b** shows that the incidence rate of NAFLD decreased with the increase of quartile of PRL concentration in female patients (*P*—trend = 0.025)

A gradual decrease in the prevalence of NAFLD was noted in both genders along with the increased quartile of PRL (Q1, 37.0%; Q2, 25.9%; Q3, 25.9%; Q4, 13.2%; *P* = 0.046 in males; Q1, 25.7%; Q2, 11.4%; Q3, 8.6%; Q4, 2.9%; *P* = 0.025 in females) (Fig. 2).

### The risk of NAFLD significantly decreased with the increment of PRL

**Fig. 3:**
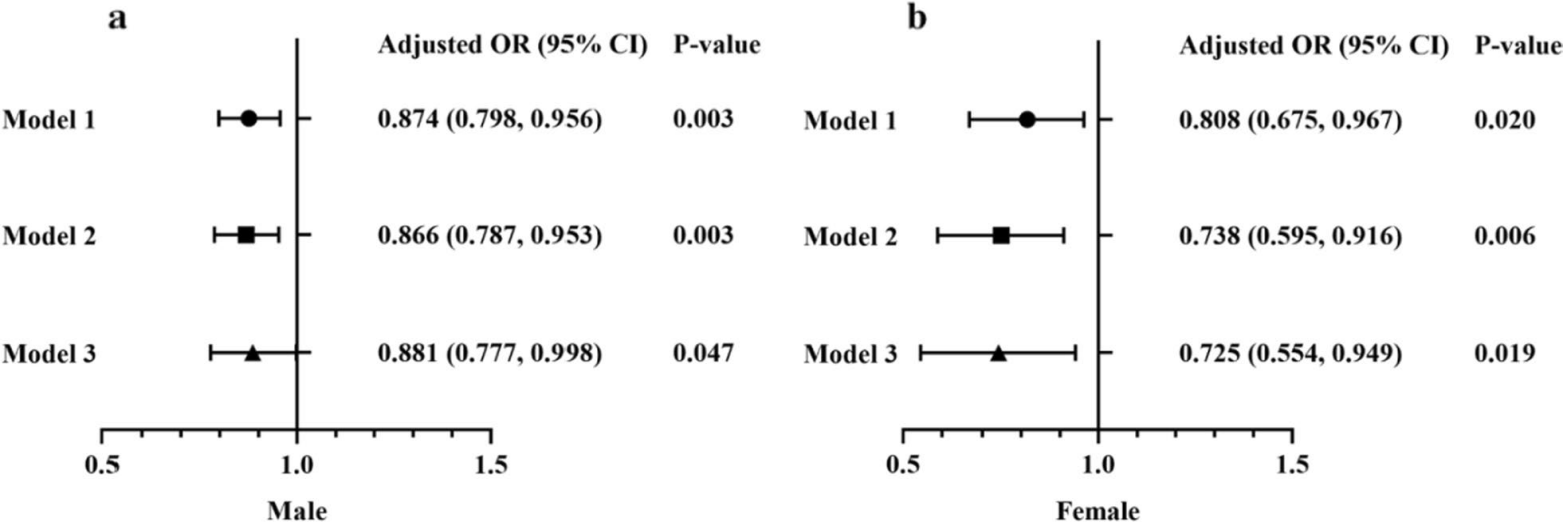
Subgroup analyses by gender (male vs. female) using logistic regression analysis. Model 1 was unadjusted, model 2 was adjusted for age and BMI, model 3 was adjusted for age, BMI, HOMA-IR, TG, TC and UA. The data are shown as the adjusted odds ratio (95% confidence interval) of serum PRL levels for the risk of non-alcoholic fatty liver disease (NAFLD) development in male patients (**Panel a**) and female patients (**Panel b**)

Three models were used to further explore the correlation between serum PRL level and risk of developing NAFLD by multivariate logistic regression analyses (Fig. 3). Serum PRL level was used as continuous variables in different sex subgroups to analyze the associations between PRL level and NAFLD development. Model 1 was unadjusted, Model 2 was adjusted for age and BMI, Model 3 was further adjusted according to age, BMI, HOMA-IR, TG, TC and UA.

Among male patients, per 1 ug/L increase in serum PRL level was associated with statistically significant effect in Model 1 [OR, 0.874 (0.798, 0.956), *P* = 0.003], Model 2 [OR, 0.866 (0.787, 0.953), *P* = 0.003] and Model 3 [OR, 0.881 (0.777, 0.998), *P* = 0.047]. Similarly in female patients, per 1 ug/L increase in serum PRL level was associated with statistically significant effect in Model 1 [OR, 0.808 (0.675, 0.967), *P* = 0.020], Model 2 [OR, 0.738 (0.595, 0.916), *P* = 0.006] and Model 3 [OR, 0.725 (0.554, 0.949), *P* = 0.019]. The inverse associations between serum PRL levels and the risk of developing NAFLD were consistent in both male and female patients.

## Discussion

This is the first longitudinal cohort study to evaluate the cause-effect relationship between serum PRL concentrations and NAFLD occurrence. After a median of 32 month follow-up, we found that serum PRL levels at baseline were significantly lower in patients who progressed to NAFLD compared with those who did not in both gender subgroups. After dividing patients into four groups according to the quartile of basal serum PRL levels, the prevalence of NAFLD decreased significantly in both genders as the quartiles of serum PRL levels increased. In multivariate logistic regression, serum PRL levels remained significantly and negatively associated with incident NAFLD after accounting for other known risk factors.

To date, several studies have examined the negative association between PRL and NAFLD, which were mainly cross-sectional and still lack longitudinal studies of causation. Chen et al. [19] reported that lower serum PRL concentrations were significantly associated with increased risk of hepatic steatosis in males with chronic hepatitis B, after adjusting for age, WC, FBG, TGs, hypertension, diabetes, and HBeAg-positive status. Consistent with that study, we also found that PRL is a protective factor against NAFLD development. However, their study was conducted only in patients with hepatitis B and included only the male population. Whereas Cuiling Zhu et al. [20] founded that high-normal serum PRL may act as a protective factor for MAFLD and hepatic fibrosis only in females with T2DM in a cross-sectional study. We previously have reported inverse relationship between serum PRL concentrations and the presence of NAFLD in a cross-sectional study [16]. Besides, we established a mathematic model for facilitating and effectively diagnosing the presence and severities of NAFLD in males and females, which also demonstrated a potential value of PRL in NAFLD [21]. To our knowledge, this study is the first to reveal that basal serum PRL level is an independent predictor for the occurrence of NAFLD in gender subgroups from a longitudinal cohort study.

PRL, recently identified as a metabolic hormone, plays a critical role in the regulation of lipid metabolism [22]. Previous studies reported that high-normal PRL concentrations were correlated with improved visceral adipocyte hypertrophy [23], adipose tissue quantity and function [24]. In adipose tissue, prolactin maintains metabolic homeostasis of adipose tissue by regulating adipose lipid metabolism, promoting the formation of new adipocytes and preventing fat cell hypertrophy [25–28]. As the largest internal organ responsible for metabolic homeostasis, the liver expresses a high level of PRLR in both rodents and human beings [29, 30]. Our previous research indicated that PRL itself may play a direct biological role in NAFLD occurrence [16]. When circulating PRL levels were reduced in patients with NAFLD, the human hepatic PRLR expression was downregulated concomitantly, and PRL intervention could enhance the expression of PRLR in HepG2 cells. PRL/PRLR might protect the liver from accumulation of lipids by inhibiting CD36 in hepatocytes [31]. It was also observed that PRL/PRLR could reduce stearoyl–coenzyme A desaturase 1 (SCD1) expression in various hepatic cell lines and animal models, which is the rate-limiting enzyme in monounsaturated fatty acid biosynthesis, thereby leading to the amelioration of hepatic steatosis [28]. These findings shed light on the role of PRL in lipid metabolism and offer a potential insight for targeting PRL for the treatment of NAFLD.

As we know, oxidative stress appears as an important pathological event during NAFLD development and the hallmark between simple steatosis and NASH manifestation. Previous studies have shown that PRL can promote the antioxidant capacity of adult retinal pigment epithelium 19 (ARPE-19) cells by reducing glutathione and block the hydrogen peroxide-induced increase in deacetylase sirtuin 2 (SIRT2) expression. RPE from PRL receptor-null mice showed increased levels of oxidative stress, SIRT2 expression and apoptosis [32]. PRL/PRLR might reduce oxidative stress in the same way and thus avoid liver damage and disease progression in NAFLD. Furthermore, unbalanced oxidative stress may lead to activation of the protease cathepsin D, which acts by cleaving prolactin with a full length 23 kDa into an angiostatic and proapoptotic 16 kDa form [33]. This process further reduces the serum PRL which exerts a protective effect on liver metabolism. Regarded as the best measure of oxidative stress in vivo, urinary 8-iso-PGF2α was an independent predictor of NAFLD. The levels of urinary 8-iso-PGF2α and serum soluble NOX2-derived peptide were increased with the severity of liver steatosis [34]. Few studies have investigated the relationship between PRL and markers of oxidative stress. In future studies, we will include these markers in the analysis to explore their role in the regulation of hepatic lipid metabolism by PRL.

Our study has some strengths including a sample based on a large population, as well as its long-term follow-up. As an estrogen-responsive pituitary hormone, PRL is dramatically elevated in females compared to males, and this difference persists in postmenopausal women compared with men [35]. The average age of the patients in our study was 56 years, and enrolled female patients were mostly in the postmenopausal state. After adjusted for age, the negative association between PRL and NAFLD still persisted in different sexes. Most importantly, it is the first study to investigate the longitudinal relationship of PRL levels with the risk of NAFLD. In addition to those already mentioned, the study has several potential limitations. First, our study was retrospective. A large, prospective study to explore whether the conclusions are consistent across age groups is needed in the future. Second, NAFLD was assessed using a non-invasive method in our study, but not liver biopsy, which is well-known as the gold standard for diagnosing NAFLD [36]. However, invasive liver biopsies in the general population are ethically impossible. In addition, abdominal ultrasound has been validated to be accurate and widely applicable for clinical examination.

In conclusion, our findings for the first time suggest the cause-effect relationship of basal serum PRL levels with incident of NAFLD, which provides a useful marker for early detection of persons at risk for NAFLD.

## Supplementary Information

**Additional file 1: Table S1.** Clinical characteristic of patients at baseline and at follow-up stratified by NAFLD status in both genders. **Table S2.** Basal clinical parameters of patients divided with the quartile of prolactin (PRL).

## Data Availability

Some or all datasets generated during and/or analyzed during the current study are not publicly available but are available from the corresponding author on reasonable request.

## Abbreviations

NAFLD: Non-alcoholic fatty liver disease
PRL: Prolactin
T1DM: Type 1 diabetes mellitus
BMI: Body mass index
WC: Waist circumference
SBP: Systolic blood pressure
DBP: Diastolic blood pressure
FBG: Fasting blood glucose
FINS: Fasting insulin
HOMA: Homeostasis model assessment
TG: Triglycerides
TC: Total cholesterol
HDL-C: High-density lipoprotein cholesterol
LDL-C: Low-density lipoprotein cholesterol
ALT: Alanine aminotransferase
AST: Aspartate transaminase
Cr: Creatinine
UA: Uric acid
eGFR: Estimated glomerular filtration rate
TSH: Thyroid stimulating hormone
SCD1: Stearoyl–coenzyme A desaturase 1
