# Supplementary material for: Lower serum PRL is associated with the development of non-alcoholic fatty liver disease: a retrospective cohort study
Source: BMC Gastroenterol. 2022 Dec 16;22:523. doi: 10.1186/s12876-022-02619-w (PMC9758822; doi:10.1186/s12876-022-02619-w)
Supplement: Supplementary file 1 — Additional file 1: Table S1. Clinical characteristic of patients at baseline and at follow-up stratified by NAFLD status in both genders. Table S2. Basal clinical parameters of patients divided with the quartile of prolactin (PRL). [file 12876_2022_2619_MOESM1_ESM.docx]

**Lower serum PRL is associated with the development of non-alcoholic fatty liver disease: A** **Retrospective Cohort Study**

**Short title: PRL associated with NAFLD**

**BMC Gastroenterology**

Ping Xu^1,2,3,*^, MD; Ye Zhu^2,3,*^, MD; Xinlu Ji^2,3^, MD; Huayang Ma^2,3^, MD; Pengzi Zhang^2,3,#^, MD, PhD; Yan Bi^1,2,3,#^, MD, PhD.

*These authors contributed equally to this work and should be regarded as co-first authors.

#These authors are co-corresponding authors.

**Affiliations**

1 Department of Endocrinology, Nanjing Drum Tower Hospital Clinical College of Nanjing Medical University, Nanjing, China

2 Department of Endocrinology, Drum Tower Hospital affiliated to Nanjing University Medical School, Branch of National Clinical Research Centre for Metabolic Diseases, Nanjing, China

3 Endocrine and Metabolic Disease Medical Center, Drum Tower Hospital affiliated to Nanjing University Medical School

**Corresponding authors**

Yan Bi,

Affiliations:

1 Department of Endocrinology, Nanjing Drum Tower Hospital Clinical College of Nanjing Medical University, Nanjing, China

2 Department of Endocrinology, Drum Tower Hospital affiliated to Nanjing University Medical School, Branch of National Clinical Research Centre for Metabolic Diseases, Nanjing, China

3 Endocrine and Metabolic Disease Medical Center, Drum Tower Hospital affiliated to Nanjing University Medical School

Telephone: 862568182302

E-mail: [biyan@nju.edu.cn](mailto:biyan@nju.edu.cn)

Pengzi Zhang,

Affiliations:

1 Department of Endocrinology, Drum Tower Hospital affiliated to Nanjing University Medical School, Branch of National Clinical Research Centre for Metabolic Diseases, Nanjing, China

2 Endocrine and Metabolic Disease Medical Center, Drum Tower Hospital affiliated to Nanjing University Medical School

Telephone: 862568182302

E-mail: [zhang_pengzi@163.com](mailto:zhang_pengzi@163.com)

Yan Bi (Orcid ID: 0000-0003-3914-7854)

**Supplementary Table 1. Clinical characteristic of patients at baseline and at follow-up stratified by NAFLD status in both genders.**

| **Variables** | **Male** | | | | | | **Female** | | | | |  |
| --- | --- | --- | --- | --- | --- | --- | --- | --- | --- | --- | --- | --- |
|  | **Non-NAFLD** | | **P-value** | **NAFLD** | | **P-value** | **Non-NAFLD** | | **P-value** | **NAFLD** | | **P-value** |
|  | **Baseline** | **Follow-up** |  | **Baseline** | **Follow-up** |  | **Baseline** | **Follow-up** |  | **Baseline** | **Follow-up** |  |
| **BMI (kg/m^2^)** | 23.84(22.05,25.40) | 23.41(22.02,24.86) | 0.022 | 24.22(22.35,26.23) | 25.46(23.45,27.53) | <0.001 | 23.75(20.45,26.59) | 23.61(20.81,25.63) | 0.006 | 26.56(23.46,29.40) | 27.34(22.17,28.79) | 0.438 |
| **WC (cm)** | 90(85,95) | 90(85,96) | 0.023 | 93(86,98) | 94(88,100) | 0.007 | 83(78,96) | 84(78,92) | 0.368 | 91(79,99) | 90(80,104) | 0.919 |
| **SBP (mmHg)** | 133(120,144) | 136(121,145) | 0.956 | 130(118,139) | 136(122,146) | 0.068 | 133(121,148) | 133(120,144) | 0.648 | 126(104,149) | 121(114,142) | 0.981 |
| **DBP (mmHg)** | 78(71,86) | 79(72,87) | 0.410 | 78(71,89) | 84(76,93) | 0.010 | 78(69,86) | 75(68,83) | 0.097 | 70(63,91) | 77(63,87) | 0.943 |
| **HbA1c (%)** | 7.90(6.75,10.00) | 7.70(6.75,9.50) | 0.457 | 7.60(6.30,10.90) | 7.40(6.40,8.45) | 0.085 | 7.05(6.00,9.23) | 6.90(5.80,8.70) | 0.445 | 8.50(5.50,10.90) | 7.20(5.98,9.35) | 0.753 |
| **FBG (mmol/L)** | 7.08(5.58,8.94) | 6.98(5.77,8.74) | 0.584 | 6.97(5.47,8.85) | 7.29(5.83,8.84) | 0.828 | 5.98(4.89,8.39) | 6.11(4.82,7.48) | 0.677 | 6.44(4.68,10.60) | 7.77(5.29,9.25) | 0.586 |
| **HOMA-IR** | 1.47(0.81,3.00) | 1.51(0.83,2.66) | 0.260 | 2.04(1.42,3.20) | 2.68(1.63,4.52) | 0.202 | 1.84(1.01,3.97) | 1.42(0.95,2.39) | 0.005 | 1.79(1.21,3.98) | 1.83(1.38,2.74) | 0.826 |
| **TG (mmol/L)** | 1.07(0.77,1.50) | 1.01(0.72,1.27) | 0.002 | 1.53(1.03,2.04) | 1.63(1.23,2.32) | 0.469 | 1.03(0.68,1.42) | 0.84(0.65,1.22) | 0.010 | 1.25(0.85,1.90) | 1.15(0.88,2.49) | 0.932 |
| **TC (mmol/L)** | 4.04(3.56,4.80) | 3.87(3.30,4.65) | 0.021 | 4.45(3.59,5.48) | 4.30(3.49,5.48) | 0.606 | 4.43(3.76,5.22) | 4.26(3.54,5.01) | 0.053 | 4.46(4.15,5.46) | 4.21(3.82,5.35) | 0.173 |
| **HDL-C (mmol/L)** | 1.07(0.84,1.28) | 1.14(0.95,1.44) | <0.001 | 0.97(0.84,1.21) | 1.00(0.81,1.19) | 0.892 | 1.19(0.98,1.57) | 1.42(1.10,1.72) | <0.001 | 1.15(1.05,1.37) | 1.12(0.98,1.45) | 0.426 |
| **LDL-C (mmol/L)** | 2.30(1.78,2.80) | 2.15(1.61,2.78) | 0.231 | 2.55(1.77,3.21) | 2.71(1.93,3.45) | 0.243 | 2.47(1.98,3.04) | 2.36(1.81,3.06) | 0.150 | 2.77(2.26,3.38) | 2.15(1.74,3.61) | 0.191 |
| **ALT (U/L)** | 17.60(13.85,24.23) | 17.70(12.78,22.80) | 0.181 | 17.40(14.05,28.00) | 20.00(16.05,25.85) | 0.533 | 17.80(13.70,24.80) | 14.40(10.85,19.50) | <0.001 | 23.90(13.50,33.25) | 20.40(13.80,28.20) | 0.426 |
| **AST (U/L)** | 17.20(14.90,20.50) | 17.60(15.08,21.03) | 0.394 | 18.60(14.15,22.05) | 17.40(14.60,21.80) | 0.739 | 18.90(15.70,22.10) | 17.70(14.50,22.60) | 0.341 | 17.80(14.50,23.85) | 17.90(14.90,21.10) | 0.955 |
| **Cr (umol/L)** | 66.00(60.00,76.00) | 66.00(59.00,79.00) | 0.924 | 65.00(58.50,74.00) | 65.50(59.00,74.50) | 0.060 | 50.00(43.00,57.20) | 51.00(44.00,57.50) | 0.273 | 48.00(40.50,57.50) | 48.00(42.00,59.00) | 0.636 |
| **UA (umol/L)** | 318.50(267.25,372.00) | 335.00(288.00,399.25) | 0.001 | 328.00(287.50,412.50) | 366.00(316.00,457.50) | 0.003 | 270.00(226.50,312.75) | 272.00(243.00,322.50) | 0.064 | 290.00(247.00,331.50) | 309.00(240.00,371.00) | 0.201 |
| **eGFR (ml/min/1.73m^2^)** | 116.46(100.05,130.16) | 114.70(93.40,130.20) | 0.408 | 116.30(99.24,140.18) | 114.30(101.10,128.40) | 0.060 | 116.69(103.09,142.47) | 119.10(99.68,151.10) | 0.563 | 130.61(100.99,168.35) | 126.50(94.80,148.05) | 0.328 |
| **TSH (mIU/L)** | 1.78(1.34,2.60) | 1.76(1.26,2.59) | 0.769 | 1.83(1.13,2.63) | 1.65(1.11,2.55) | 0.813 | 2.20(1.44,3.19) | 2.22(1.50,3.08) | 0.493 | 1.98(1.45,3.22) | 2.31(1.51,3.00) | 0.352 |
| **PRL (ug/L)** | 9.13(6.92,12.50) | 7.27(5.49,9.08) | <0.001 | 7.35(5.48,10.60) | 6.37(4.83,8.72) | 0.023 | 9.01(6.31,11.60) | 7.36(5.42,10.70) | <0.001 | 5.66(4.67,9.03) | 5.89(3.90,8.27) | 0.407 |

The data are expressed as mean ± standard deviation or median (interquartile range).

NAFLD, non-alcoholic fatty liver disease; BMI, body mass index; WC, waist circumference; SBP, systolic blood pressure; DBP, diastolic blood pressure; FBG, fasting blood glucose; HOMA, homeostasis model assessment; TG, triglycerides; TC, total cholesterol; HDL-C, high-density lipoprotein cholesterol; LDL-C, low-density lipoprotein cholesterol; ALT, alanine aminotransferase; AST, aspartate transaminase; Cr, creatinine; UA, uric acid; eGFR, estimated glomerular filtration rate; TSH, thyroid stimulating hormone; PRL, prolactin.

**Supplementary Table 2. Basal clinical parameters of patients divided with the quartile of prolactin (PRL).**

| **Variables** | **Male** | | | | | **Female** | | | | |
| --- | --- | --- | --- | --- | --- | --- | --- | --- | --- | --- |
|  | **Q1**  **n=54** | **Q2**  **n=54** | **Q3**  **n=54** | **Q4**  **n=53** | **P-value** | **Q1**  **n=35** | **Q2**  **n=35** | **Q3**  **n=35** | **Q4**  **n=35** | **P-value** |
| **Age (year)** | 57(48,66) | 58(50,66) | 56(49,64) | 57(49,64) | 0.983 | 61(55,64) | 56(51,64) | 54(48,66) | 45(32,60) | 0.008 |
| **BMI (kg/m^2^)** | 23.69(22.15,25.27) | 24.22(22.44,25.56) | 23.90(22.28,25.63) | 23.86(21.91,25.95) | 0.737 | 24.48(21.15,28.00) | 24.14(20.93,26.56) | 22.49(19.88,28.72) | 24.65(21.09,33.06) | 0.598 |
| **WC (cm)** | 89(84,94) | 89(83,96) | 91(87,96) | 91(86,96) | 0.619 | 85(78,96) | 86(78,96) | 81(78,93) | 88(79,106) | 0.527 |
| **Follow-up (month)** | 26(20,35) | 34(22,46) | 36(24,50) | 35(21,49) | 0.198 | 22(12,36) | 24(15,34) | 43(17,55) | 34(16,45) | 0.022 |
| **Diabetes, n (%)** | 53 (98.1%) | 51 (94.4%) | 53 (98.1%) | 51 (96.2%) | 0.653 | 30 (85.7%) | 28 (80.0%) | 25 (71.4%) | 25 (71.4%) | 0.405 |
| **Hypertension, n (%)** | 33 (61.1%) | 28 (51.9%) | 27 (50.0%) | 26 (49.1%) | 0.576 | 16 (45.7%) | 22 (62.9%) | 16 (45.7%) | 18 (51.4%) | 0.433 |
| **Hyperlipidemia, n (%)** | 22 (40.7%) | 14 (25.9%) | 16 (29.6%) | 11 (20.8%) | 0.134 | 12 (34.3%) | 10 (28.6%) | 9 (25.7%) | 7 (20.0%) | 0.598 |
| **SBP (mmHg)** | 132(122,142) | 134(122,145) | 132(119,141) | 131(117,143) | 0.805 | 132(119,145) | 132(121,148) | 131(120,147) | 137(121,152) | 0.541 |
| **DBP (mmHg)** | 80(72,89) | 79(71,89) | 76(70,86) | 77(71,84) | 0.390 | 73(63,86) | 76(67,85) | 74(63,84) | 83(71,91) | 0.044 |
| **HbA1c (%)** | 7.80(6.80,10.95) | 7.50(6.50,9.70) | 8.50(6.43,10.13) | 7.50(6.60,9.78) | 0.618 | 8.50(6.10,10.50) | 7.30(6.00,10.00) | 6.70(5.73,7.83) | 6.60(5.40,9.20) | 0.102 |
| **FBG (mmol/L)** | 7.74(5.94,10.31) | 7.31(5.94,8.62) | 7.00(5.35,8.89) | 6.33(4.99,8.14) | 0.038 | 6.95(5.46,10.43) | 5.81(4.85,8.12) | 6.44(4.76,8.69) | 5.29(4.66,7.33) | 0.101 |
| **HOMA-IR** | 1.73(0.98,2.71) | 2.26(1.32,3.20) | 1.73(0.70,3.31) | 1.19(0.70,2.39) | 0.147 | 1.79(1.05,2.84) | 1.27(0.76,2.75) | 2.77(1.11,5.22) | 2.21(1.14,6.16) | 0.051 |
| **TG (mmol/L)** | 1.12(0.77,1.56) | 1.34(0.87,1.90) | 1.17(0.72,1.98) | 1.06(0.88,1.54) | 0.480 | 0.96(0.69,1.39) | 1.14(0.77,1.44) | 1.01(0.63,1.42) | 1.12(0.68,1.66) | 0.793 |
| **TC (mmol/L)** | 4.32(3.61,5.23) | 4.21(3.57,5.24) | 3.93(3.53,4.56) | 3.93(3.55,4.85) | 0.152 | 4.48(4.12,5.40) | 4.57(3.82,5.38) | 4.36(3.76,5.28) | 4.36(3.56,5.09) | 0.724 |
| **HDL-C (mmol/L)** | 1.04(0.88,1.24) | 1.06(0.81,1.24) | 0.99(0.81,1.31) | 1.11(0.84,1.30) | 0.873 | 1.23(1.02,1.58) | 1.20(1.07,1.45) | 1.34(1.09,1.76) | 1.06(0.92,1.26) | 0.018 |
| **LDL-C (mmol/L)** | 2.53(1.90,3.35) | 2.44(1.95,2.99) | 2.04(1.62,2.71) | 2.11(1.66,2.70) | 0.030 | 2.67(2.26,3.22) | 2.45(2.02,3.31) | 2.47(1.76,2.93) | 2.38(1.86,3.14) | 0.348 |
| **ALT (U/L)** | 17.40(13.65,22.55) | 17.30(14.13,24.73) | 20.80(14.20,26.50) | 17.10(12.90,24.60) | 0.583 | 17.05(13.65,23.50) | 17.90(12.75,26.25) | 19.10(13.70,25.60) | 17.30(13.65,25.15) | 0.978 |
| **AST (U/L)** | 16.10(14.45,19.75) | 17.70(14.28,21.58) | 18.75(15.68,21.40) | 17.50(14.00,21.80) | 0.498 | 17.80(15.55,20.10) | 18.90(15.10,23.80) | 18.90(17.20,21.70) | 19.40(14.08,22.93) | 0.688 |
| **Cr (umol/L)** | 64.00(58.50,72.00) | 68.00(60.00,76.00) | 67.00(61.00,77.00) | 66.00(59.00,75.00) | 0.392 | 52.50(43.00,56.25) | 50.00(42.50,59.00) | 49.00(44.00,61.25) | 49.50(42.50,56.25) | 0.935 |
| **UA (umol/L)** | 294.00(269.50,342.00) | 330.00(279.50,401.00) | 325.00(273.50,386.50) | 325.00(266.00,382.00) | 0.187 | 258.00(218.00,307.50) | 270.00(237.00,301.50) | 252.00(207.75,300.25) | 301.00(262.25,370.00) | 0.011 |
| **eGFR (ml/min/1.73m^2^)** | 117.55(101.65,138.32) | 113.63(96.32,133.77) | 110.49(98.38,129.19) | 118.44(108.00,132.60) | 0.593 | 106.99(102.63,137.46) | 118.10(102.75,152.40) | 126.65(105.01,140.48) | 131.56(103.37,148.41) | 0.630 |
| **TSH (mIU/L)** | 1.65(1.27,2.16) | 1.73(1.02,2.45) | 1.80(1.19,2.60) | 2.01(1.53,3.01) | 0.093 | 2.34(1.43,3.20) | 2.04(1.42,2.89) | 2.06(1.36,3.17) | 2.36(1.60,3.69) | 0.492 |
| **PRL (ug/L)** | 5.33(4.64,6.09) | 7.64(7.06,8.14) | 10.19(9.29,11.01) | 14.7(13.01,18.70) | <0.001 | 4.90(3.97,5.40) | 7.16(6.45,7.90) | 9.81(9.34,10.52) | 16.80(12.50,23.20) | <0.001 |

The data are expressed as mean ± standard deviation or median (interquartile range).

NAFLD, non-alcoholic fatty liver disease; BMI, body mass index; WC, waist circumference; SBP, systolic blood pressure; DBP, diastolic blood pressure; FBG, fasting blood glucose; HOMA, homeostasis model assessment; TG, triglycerides; TC, total cholesterol; HDL-C, high-density lipoprotein cholesterol; LDL-C, low-density lipoprotein cholesterol; ALT, alanine aminotransferase; AST, aspartate transaminase; Cr, creatinine; UA, uric acid; eGFR, estimated glomerular filtration rate; TSH, thyroid stimulating hormone; PRL, prolactin.
